# Supplementary figures and images for: Effects of Hypocalcemic Vitamin D Analogs in the Expression of DNA Damage Induced in Minilungs from hESCs: Implications for Lung Fibrosis
Source: Int J Mol Sci. 2022 Apr 28;23(9):4921. doi: 10.3390/ijms23094921 (PMC9104735; doi:10.3390/ijms23094921)

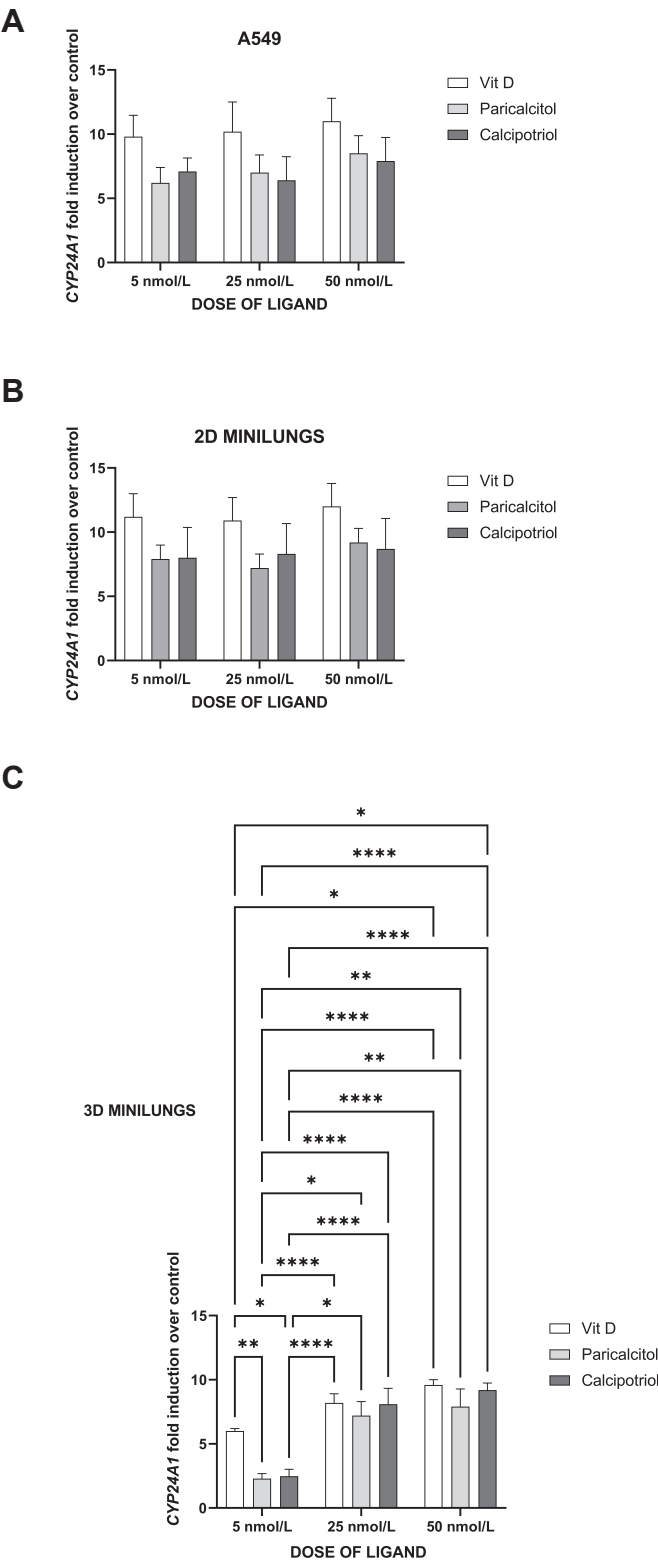

Supplement: Supplementary file 1 [file ijms-23-04921-s001.zip › ijms-1693199-supplementary.pdf]
